# Pre-cleaning of hair is not beneficial in LA-ICP-MS studies of chronic metal exposure

**DOI:** 10.1371/journal.pone.0289635

**Published:** 2023-08-10

**Authors:** Gwendolyn K. David, Andrew H. Hunter, Karine H. Moromizato, Charlotte M. Allen, Rebecca Wheatley, Frank A. von Hippel, Amanda C. Niehaus, Robbie S. Wilson

**Affiliations:** 1 School of Biological Sciences, The University of Queensland, St Lucia, QLD, Australia; 2 Central Analytical Research Facility, Queensland University of Technology, Brisbane, QLD, Australia; 3 Department of Community, Environment & Policy, Mel & Enid Zuckerman College of Public Health, University of Arizona, Tucson, Arizona, United States of America; Harvard School of Public Health, UNITED STATES

## Abstract

Chronic exposure to toxic metals is a serious global health concern. However, population-wide biomonitoring is costly and carries several sampling constraints. Though hair sampling can be a useful way to assess environmental exposure, external contamination is a long-standing concern, and a pre-cleaning step prior to metal quantification has long been recommended despite a lack of evidence for its efficacy. In this study, we quantified the spatial distribution of 16 elements in unwashed human hair samples using Laser Ablation Inductively Coupled Plasma Mass Spectrometry (LA-ICP-MS), then tested how two common pre-cleaning treatments (Triton-ethanol, Triton-nitric acid) affected metal content in external and interior layers of hair using LA-ICP-MS. We show that elements differ in their spatial distribution across hair and that pre-cleaning is not consistent in its effect on element concentrations and decreases interior concentrations of some elements. We demonstrate that differences among individuals can be quantified reliably with LA-ICP-MS analysis of interior concentrations of unwashed hair. Our study tests the widespread notion that pre-cleaning is essential in analyses of hair for environmental exposure to metals, and examines the benefits of a unified approach to analysis of metals in hair using LA-ICP-MS.

## Introduction

Chronic exposure to toxic elements is a serious health concern [1, 2], but population-wide monitoring of individual exposures is costly and logistically challenging—particularly for common sampling mediums of blood and urine [3, 4]. Hair samples provide a useful screening tool for assessing metal exposure because collection of hair is quick and non-invasive, modern technologies can make assessment affordable, and the highly stable matrix of hair requires only basic storage conditions [3, 5–8]. Metallic elements are metabolically incorporated in scalp hair as it grows so that long-term exposure (i.e. months) can be quantified using hair growth rates (~1cm/month, range = 0.82–1.28cm/month) [9–11]. This provides advantages compared with blood and urine, which allow assessment of only short-term windows of exposure (i.e. hours or days) [3]. Hair used for environmental toxicology studies typically undergoes a lengthy pre-cleaning process to remove external contaminants before analysis [3], even though the presence of external contamination is questionable and the effectiveness of pre-cleaning demands evidence [7, 11, 12].

Evidence of external contamination of hair come from studies of chronic exposure to toxicants [13–15] and *in-vitro* experimental work [16–18]. For example, children had detectable drug concentrations in hair associated with illegal drug use by parents [15]—although distinguishing external contamination from metabolically-derived accumulation is difficult [7, 14, 15]. Whilst experimental studies have shown that artificial contamination can occur and be significantly reduced by pre-cleaning hair prior to quantifying metal or drug concentrations [13, 16–18], questions remain about the generalizability of conclusions drawn from these *in-vitro* studies [14]. Even if one were to partition external and systemic metal accumulation in hair, both sources reflect an individual’s exposure to environmental toxicants and both are relevant measures. As such, the widely-used pre-cleaning step may eliminate useful information about chronic exposure to toxic pollutants.

Hair samples are typically pre-cleaned with a non-ionic detergent and solvent (e.g., Triton X-100, nitric acid, ethanol, acetone, etc.) to remove external contamination prior to acid digestion into solution; elemental composition of samples is then usually quantified with Inductively Coupled Plasma Mass Spectrometry (ICP-MS) [7, 18–20]. However, solution-based ICP-MS analysis does not differentiate metal accumulation among layers or regions of hair, because the whole hair sample is homogenised through acid digestion before undergoing quantification [3, 7, 18, 21, 22]. Furthermore, the pre-cleaning step is contentious as it carries a risk of stripping metabolically-incorporated metal from the hair interior [7] or promoting migration of external contaminants into the hair strand [13].

With an alternative technique, Laser Ablation Inductively Coupled Plasma Mass Spectrometry (LA-ICP-MS), detailed microanalyses (+/- 1μm) can be used to quantify variation in interior metal content across and along the length of a complete hair strand, offering detailed spatial data that traditional ICP-MS cannot [23, 24]. For example, LA-ICP-MS has been used to quantify the uptake of metals from diet or medication over several months by analysing single hair strands from root to tip [5, 21, 25]. LA-ICP-MS is a widely applicable technique that has enabled detailed bio-imaging of metals in a range of other tissues, including the brain, liver, eye, kidney, tooth, bone, blood, claw, feather, nail, and scale; it has even been applied to mummified remains [11, 24, 26–34]. LA-ICP-MS requires far less sample than ICP-MS (i.e. a few strands versus ~200 strands) [7, 22, 27], which is particularly important when sampling scalp hair from children or in culturally sensitive societies. After the initial outlay for equipment, analyses of samples can be much cheaper by LA-ICP-MS than by ICP-MS. The main criticism of LA-ICP-MS relates to calibration as compared to calibration of solutions from a range of concentrations that encompass the sample for each element. LA-ICPMS relies on a one-point calibration from a well characterized reference material, and use of an internal standard element [35]. The quantification of an element, Mn for example, does rely on knowing the internal standard element concentration, in this case carbon. This methodology is widely used and produces results for other monitor reference materials at better than 10% and many better than 5%. LA-ICP-MS may also be useful as an alternative to pre-washing—namely, it might be used to ablate the external layer of hair samples before the interior is quantified [11, 36], saving considerable time and cost.

To investigate this potential, we collected hair samples from 21 Indigenous people residing near or far from a mine in Australia. We assessed the distribution of 16 elements in unwashed hair using LA-ICP-MS (spot drilling and line scans) and then tested how two pre-cleaning treatments (Triton-ethanol or Triton-nitric acid) affected metal content in external and interior layers of hair using LA-ICP-MS (line scans). We provide recommendations for the use of LA-ICP-MS in future studies of environmental toxicology.

## Method

### Ethics

This study is part of a research project investigating metal exposures in Indigenous people. Ethical approval was granted by The Northern Territory Menzies School of Health (Ref 2015–2478) and The University of Queensland Human Research Ethics Committee (Ref 2015001976). All research was performed in accordance with the *Australian Code for the Responsible Conduct of Research* (2007) and the *Values and Ethics*: *Guidelines for Ethical Conduct in Aboriginal and Torres Strait Islander Health Research* (2003). Written informed consent was obtained from all participants and/or their legal guardians.

### Sample collection and preparation

Hair samples were collected from 21 participants (age range = 5-61yrs; 11 ♀/10 ♂). Approximately 30 strands of hair were cut from each participant using stainless-steel scissors as close to the occipital region of the scalp as possible. Samples were stored in an envelope at room temperature until analysis at the Central Analytical Research Facility operated by the Institute for Future Environments at Queensland University of Technology. The colour and thickness (40–70μm) of strands varied within individuals, so hair strands were selected randomly from within each sample.

Prior to LA-ICP-MS, hair sub-samples were pre-cleaned with Triton-ethanol (EC; 3 strands) or Triton-nitric acid (NC; 3 strands), or were left unwashed (UW; 8 strands). In the EC treatment, hair samples were sonicated (15min) in 0.5% Triton X-100, rinsed three times with Milli Q water, sonicated again (15min) in 95% ethanol solution, rinsed with Milli Q water, then dried overnight at 65°C [18]. For the NC treatment, hair samples were sonicated (20min) in 0.5% Triton X-100, rinsed 5 times with Milli Q water, sonicated again (10min) in 1N nitric acid, rinsed once with 1N TMG nitric acid, rinsed 5 times with Milli Q water, then dried overnight at 65°C [18].

### Metal quantification

#### LA-ICP-MS

Following the pre-cleaning treatment, each hair strand was blown with compressed N_2_ for 20 seconds to remove any dust sitting on the hair surface, then fixed to a microscope glass slide using double-sided sticky tape. Spatial distribution of elements in hair strands, as represented by one isotope (^27^Al, ^31^P, ^43^Ca, ^53^Cr, ^55^Mn, ^57^Fe, ^59^Co, ^60^Ni, ^63^Cu, ^66^Zn, ^75^As, ^88^Sr, ^111^Cd, ^137^Ba, ^201^Hg, ^208^Pb), was quantified from two different laser ablation modes, either spot drilling or line scanning using a Triple Quadruple Inductively Coupled Plasma Mass Spectrometer (Model 8800, Agilent Technologies Inc., Santa Clara, USA) used in single quad mode and coupled with a 193nm wavelength excimer laser and a two-volume cell (ESI New Wave Research, Bozeman, USA). The operating parameters were: RF power 1300 watts, carrier gas He, carrier gas flow rate 600ml /min, torch depth 4mm, cell gas He, cell gas flow rate 500ml/min, torch injection tube diameter 2.5mm, laser energy fluence 1.5 J/cm^2^, pulse rate 6Hz, masked circular beam 50μm. The analyte list and dwell times are given in S1 Table in S1 File. ^13^C was used as the internal standard [32, 37, 38], and it was assumed to have a total Carbon content of 42 wt% derived from analysis of a sub-sample of hairs using TruSpec Micro CHNS (LECO Corporation, St Joseph, USA).

Spot drilling using LA-ICP-MS measures element concentrations *through* a hair strand by ablating numerous sequential microlayers, thus creating a cross-sectional profile. For spot drilling, 5 laser spots along the first 1cm of hair at the root-end were ablated at 0.25cm intervals (S6 Fig in S1 File), with a 40sec background signal taken between spots that were drilled for 30 seconds. Line scan LA-ICP-MS measures element concentrations *along* a hair strand by ablating layers from root to tip, thus creating a longitudinal profile from the few microns of material removed from the surface by the laser. For each line scan, the first ablation quantified metal in the “external” layer of the hair strand (estimated 10–15 μm removed, scan speed 50μm/sec), followed by a second ablation of the “interior” (another estimated 10–15 μm removed, scan speed 50μm/sec); the remaining hair was left un-ablated. For each line scan, data were averaged across 0.5cm longitudinal sections of hair strands up to 7cm in length. LA-ICP-MS data were collected and reduced using *IOLITE* software (v3). For each individual, 25 spot drills across 5 unwashed hair strands (5 per strand) and 18 line scans (3 strands for each of the two cleaning treatments plus the unwashed treatment, 2 ablations per strand) were analysed. Not all 21 individuals could be analysed for each cleaning-scan method, and those sub-samples containing only one strand were removed from analyses, leading to final sample sizes of: UW-spot (20), UW-line (19), EC-line (20) and NC-line (18).

Representative limits of detection were calculated and averaged from 30 unwashed hair strands (μg/g): Al (0.33), As (0.28), Ba (0.14), Ca (38.8), Cd (0.13), Co (0.03), Cr (0.64), Cu (0.17), Fe (4.82), Hg (0.20), Mn (0.29), Ni (0.37), P (3.38), Pb (0.21), Sr (0.14) and Zn (0.24). The external standard was un-washed, reground pelleted human hair CRM, NCS ZC81002b (China National Analysis Centre for Iron and Steel, Beijing, China) and was analysed multiple times each day (M = 12.1, range = 5–24). The external standard was analysed concurrently with the samples, in between every 10–15 hairs. As such approximately half the measures of the external standard were line scans and half were spot drilled. The mean±se recoveries across N = 97 analyses were: Al (103±2%), As (100±6%), Ba (101±1%), Ca (103±2%), Cd (112±6%), Co (112±4%), Cr (114±4%), Cu (111±5%), Fe (110±3%), Hg (100±4%), Mn (104±2%), Ni (111±5%), P (101±1%), Pb (105±2%), Sr (101±1%) and Zn (101±1%).

### Statistical analysis

To assess sources of variation in unwashed hair (LA-ICP-MS line scan data of entire strands, up to 7cm in length), the best fit linear mixed effects model (LME) was determined based on Akaike Information Criterion (AIC) and likelihood ratio tests (LR) (*R* code, S1.1 Text; S2 Table in S1 File, models tested). To understand how pre-cleaning affected each element, post-hoc Tukey contrasts with single-step adjusted p-values for multiple comparisons were calculated using a subset of LA-ICP-MS line scan data (first 2cm of strands from the root-end), with one fixed factor (Cleaning-Layer, six concentrations) and strand nested within individual as a random factor (*R* code, S1.2 Text in S1 File). To assess if pre-cleaning affected element concentrations in the same way, the best fit LME (based on AIC and LR tests) was determined using LA-ICP-MS line scan data at 2cm, with three fixed factors and their interactions (element, cleaning, layer) and strand nested within individual as a random factor (*R* code, S1.3 Text in S1 File). For all LMEs, marginal (*m*R^2^, the proportion of variance explained by the fixed factor alone) and conditional (*c*R^2^, the proportion of variance explained by both fixed and random factors combined) R^2^ values were calculated–these measures are similar to a standardised effect size statistic explaining the magnitude of an effect in LMEs, and are bound between 0 and 1, with a higher value indicating a greater effect [39]. Principal Component Analyses (PCA) in *JMP Pro* (v13.1, SAS) were used to assess the correlation in participant element concentrations across hair layers and cleaning methods (six variables). Where required, metal concentrations were transformed using an inverse hyperbolic sine function to satisfy the assumption of normality. Aside from PCAs, all statistical analyses were performed in *R* (v3.3.3) using packages *nlme* (v3.1–131), *geoR* (v1.7–5.2), *multcomp* (1.4–6) and *MuMIn* (v1.40.4). For all statistical tests, the critical alpha value was set at 0.05.

## Results

### Sources of variation in unwashed hair

**Fig 1:**
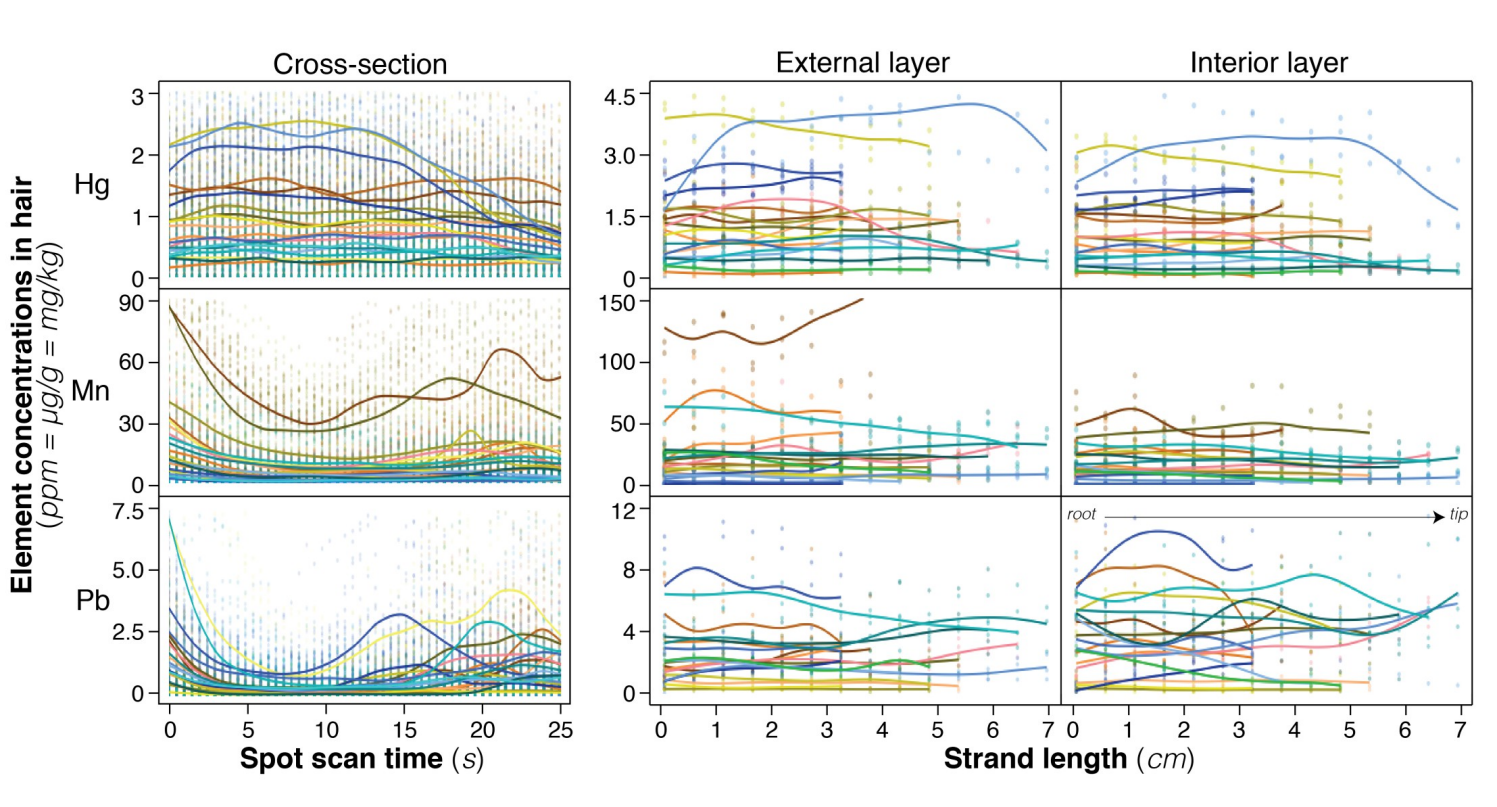
Variation in Hg, Mn and Pb of unwashed hair using LA-ICP-MS from 20 individuals. Element concentration plotted across (spot drilling, cross-section, time in seconds as a proxy for depth) and along (line scans, external & interior layers) hair strands. Raw spot data (~0.3s instantaneous concentrations) and averaged line scan data (mean across 0.5cm sections) from every hair strand are plotted, and overlaid by a smoothed cubic spline across strands for each individual (curvy lines, **λ** = 0.05). Each individual is delineated by a unique colour. For all elements see S7, S8 Figs in S1 File.

Element concentrations varied considerably in unwashed hair, with differences among individuals explaining the most variation (S2 Table in S1 File, all models *c*R^2^ v *m*R^2^, M2 v M5, LR = 3817, *P* <0.0001; Fig 1, S7, S8 Figs in S1 File). Within individuals, element concentrations differed among strands (S2 Table in S1 File, M2 v M4, LR = 334, *P* <0.0001), and between layers of hair (S2 Table in S1 File, M2 v M3, LR = 450, *P* <0.0001), illustrated by bi-modal distributions in cross-sections of hair (S2 Table in S1 File; Fig 1, S7, S8 Figs in S1 File). Whilst there was no consistent pattern in how element concentrations varied along the length of strands (S2 Table in S1 File, M1 vM2, LR <0.0001, *P* = 0.9971), some individuals displayed fluctuating concentrations (Fig 1, S7, S8 Figs in S1 File).

After accounting for variation among individuals, we found that the external layer of hair strands had greater Al, As, Ca, Co, Cu, Fe, Hg, Ni and Mn; lower Cd, Cr, P, Pb, and Zn; and around the same Ba and Sr concentrations as hair interiors (S3 Table in S1 File, UW^E^ v UW^C^). Spot and line LA-ICP-MS methods revealed elemental composition varied across hair layers, with examples of three elements (Hg, Mn, Pb) presented in Fig 1 (see S7 and S8 Figs in S1 File for all elements). In line scans over the first 2cm of hair—representing ~2 months of exposure—Hg (S3 Table in S1 File, *z* = 6.1, *P* < 0.05) and Mn (*z* = 3.4, *P* < 0.05) concentrations were greater in the external layer compared to the interior and Pb was lower in the external layer compared to the interior (*z* = -3.9, *P* < 0.05), with notable individual variation (Fig 1). In spot drilling—representing up to ~25 non-consecutive 3.5hr windows of exposure over a month—Hg was homogenous over the cross-sections of hair, with Mn and Pb greater in the external than the interior layer (Fig 1).

### Pre-cleaning treatments

**Fig 2:**
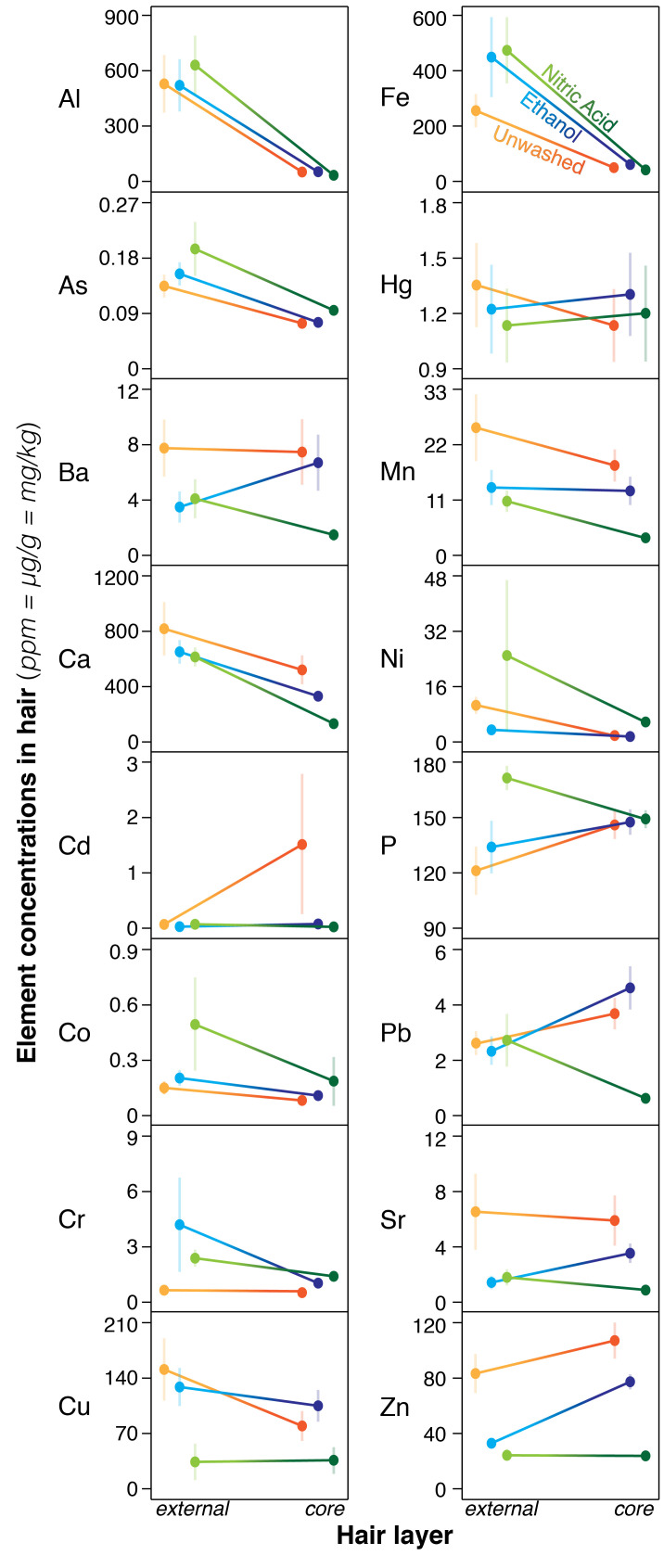
Pre-cleaning effects on hair analysed by LA-ICP-MS. Differences in element concentrations among cleaning treatments (unwashed UW = orange, ethanol EC = blue, nitric acid NC = green) and hair layers (external = lighter shade on left side, interior = darker shade on right side) for each element. Mean element concentrations across individuals (solid circles), standard errors (vertical bars) and hair-layer differences (solid gradient lines) illustrate the interactive effects among element, layer and cleaning from the best fit linear mixed effects model (S4 Table in S1 File). Note the three cleaning treatments within each layer are offset for clarity.

Because line scans covered greater exposure times compared with our spot drilling (~2 months v max. 90h), and because strand length had no effect on measurement and analyses of longer hair samples were more costly, we investigated the effect of pre-cleaning treatments using only the first 2cm of line scans. Cleaning hair prior to analysis affected external and interior layers differently, with no consistent pattern across elements (Fig 2; S4 Table in S1 File, LME, all interactions, *P*<0.0001; S9 Fig in S1 File). For Ba, Mn, and Sr, both hair cleaning methods reduced concentrations in external and interior layers of hair relative to unwashed samples (S3 Table in S1 File, all *P*<0.05). Compared to unwashed hair, cleaning hair using the EC treatment decreased interior concentrations of Ba, Ca, Mn, and Sr, and increased concentrations Co, Cr, Cu, Fe, and Hg (S3 Table in S1 File, EC^C^ v UW^C^). The EC treatment also decreased external concentrations of Ba, Ca, Cd, Hg, Mn, Ni, Pb, Sr, and Zn, and increased concentrations of As, Co, Cr, Fe, and P (S3 Table in S1 File, UW^E^ v EC^E^). Compared to unwashed hair, the NC treatment decreased interior concentrations of Al, Ba, Ca, Cd, Cu, Mn, Ni, Pb, Sr, and Zn, and increased concentrations of Cr (S3 Table in S1 File, NC^C^ v UW^C^). The NC treatment also decreased external concentrations of Ba, Cd, Cu, Hg, Mn, Ni, Pb, Sr, and Zn, and increased concentrations of Al, As, Co, Cr, Fe, and P (S3 Table in S1 File, UW^E^ v NC^E^). While there was no clear pattern of difference between the cleaning methods on external concentrations (Fig 2; S3 Table in S1 File, NC^E^ v EC^E^), the NC treatment tended to reduce interior concentrations to a greater extent than the EC treatment across most elements (S3 Table in S1 File, NC^C^ v EC^C^, negative *z*-values). Regardless of which treatment was used (EC, NC, UW), relative element concentrations among individuals were consistent (S5 Table in S1 File); for example, individuals with high Mn in hair detected by one method also showed high concentration by the other method.

## Discussion

Element concentrations varied among individuals, among hair strands within individuals, and across different layers with each hair. Pre-cleaning with either chemical significantly reduced interior concentrations of some elements and increased concentrations of others (Fig 2), which may affect measures of metabolically-incorporated metals [7]. In future studies using LA-ICP-MS, we recommend that hair samples are *not* chemically-cleaned but that the external layer of hair is ablated before using the interior as a consistent measure of individual differences in metal accumulation [11, 36].

Study participants differed in the accumulation of elements in hair, which was likely due to differences in participants’ hair growth or integrity, exposure to metals, diet, or a mixture of these factors [7, 14]. Differences in elemental distribution between external and interior layers may reflect modes of uptake—environmental exposure versus ingestion—and/or elements being laid down differently across layers during hair formation [7]. However, it is not possible to link these differences to external contamination through absorption from the environment without controlled experimental evidence [7]. Such experiments must control for ambient exposure to elements and diet as well as demographic variation over substantial time periods prior to the spatial mapping of hair samples. In the absence of such empirical confirmation, we must take care not to assume that external contamination is rife and that hair samples must be pre-cleaned. Rather, we should establish a consistent, evidence-based protocol of hair analysis that minimises spurious effects.

For several elements, pre-cleaning with ethanol or nitric acid reduced concentrations not just in the external layer, but the interior too, which could cause exposure to be underestimated. We found differences between elements in their spatial accumulation over cross-sections of hair possibly attributed to variation in deposition during growth, susceptibilities to absorption from external sources, or chemical reactions during pre-cleaning [7, 18]. Chemicals used in cleaning protocols may bind to elements in different ways, degrade hair structures, leach metals from the hair interior and deposit them in the external layer—or vice versa, or even remove them entirely [7, 13]. For example, nitric acid, which is used to completely dissolve and homogenise hair samples prior to ICP-MS, is used as a pre-cleaning chemical—yet this may partially degrade the hair structure, allowing elements to move between layers or out of the hair [7, 18].

Many argue that a pre-cleaning step is necessary for toxicology studies using hair samples [3], but we found that pre-cleaning was inconsistent in removing unconfirmed external contamination and may cause more problems than it solves, particularly if LA-ICP-MS is available. Whilst inconsistences in hair analysis methods in toxicology studies make comparisons of among-study results difficult or impossible, within-study comparisons are still valid and useful. Solution ICP-MS does have lower limits of detection than LA-ICP-MS [22] (because count levels can be sustained for minutes not seconds), and therefore may be preferable if low concentrations of elements are of interest. However, if the traditional solution ICP-MS method is used, care should be taken if choosing to use a chemical pre-cleaning step, as we have shown that it affects elements differently. Results must be interpreted with this in mind. Should a pre-cleaning step be used, element concentrations in both the hair *and* pre-cleaning solution residue can be quantified to provide an indication of washing effectiveness and/or probability of external contamination [14].

We show that LA-ICP-MS is a useful tool for population-wide monitoring of high or chronic exposure to toxic elements. Yet to avoid inconsistences in methods [18] and pre-cleaning effects across elements, we propose several recommendations for the use of LA-ICP-MS in toxicology studies of hair. First, a minimum of three hair strands should be analysed for each individual, given that we found significant within-individual strand effects for some people (S10 Fig in S1 File, error bars). Second, if line scans are utilised, the top layer of hair (~10–15 microns) can be removed with an initial ablation [11, 36], thereby avoiding inconsistences associated with pre-cleaning and possible external contamination; a second ablation can then focus on the interior region as a reliable estimate of exposure, or use both interior and external layer measures as separate estimates of exposure. However, if spot drilling is used, we recommend sampling considerably more locations along the strand at smaller intervals than we sampled here, to capture the potentially high variation in exposure over time [5, 21]. Finally, we assessed exposures using line scans that were averaged over 0.5cm sections of hair, which translated to ~2 weeks growth intervals. Though this simplified statistical analyses, we sacrificed detailed information on daily exposure. Therefore, whilst the length of hair strands analysed may depend upon on the time-frame of interest, research question and cost, we recommend not summarising data prior to statistical analyses as these are likely to reflect true variation in environmental exposure and should be taken into account.

To our knowledge, our study is the first to test the effect of pre-cleaning hair samples when using LA-ICP-MS to estimate environmental exposure to toxicants. We show that elements differ in how they are spatially distributed in hair and how they respond to a pre-cleaning step. Furthermore, we demonstrate that differences between individuals can be reliably quantified using interior region concentrations of unwashed hair. We provide recommendations for future hair analysis using LA-ICP-MS in the hopes of improving the quality and comparability of environmental toxicological measures.

## Supporting information

S1 File Supplementary material. R-code, statistical tables, and figures.
(DOCX)

S2 File Inclusivity in global research. (DOCX)
