# Supplementary material for: Pre-cleaning of hair is not beneficial in LA-ICP-MS studies of chronic metal exposure
Source: PLoS One. 2023 Aug 10;18(8):e0289635. doi: 10.1371/journal.pone.0289635 (PMC10414646; doi:10.1371/journal.pone.0289635)
Supplement: S1 File — R-code, statistical tables, and figures. (DOCX) [file pone.0289635.s001.docx]

Supplementary Material

Pre-cleaning of hair is not beneficial in LA-ICP-MS studies of chronic metal exposure

GK David, A Hunter, KH Moromizato, C M Allen, R Wheatley, FA von Hippel, AC Niehaus and RS Wilson

## **Supplementary Text S1**. R-code for statistical analyses

#### Text S1.1 R-code, analyses of unwashed hair variation

# Load packages

library(nlme)

library(geoR)

library(multcomp)

library(MuMIn)

#  Re-level so that “intercept” is the interior & subset to unwashed data only

dat$layer <- relevel(dat$layer, ref = "interior")

UWdat <- subset(dat, cleaning == "UW")

# Inverse Hyperbolic Sine (IHS) transformation of concentrationZ

IHS.loglik <- function(theta,x){

  IHS <- function(x, theta){

    asinh(theta * x)/theta

  }

    n <- length(x)

  xt <- IHS(x, theta)

    log.lik <- -n*log(sum((xt - mean(xt))^2))- sum(log(1+theta^2*x^2))

  return(log.lik)

}

## Optimise for concentrationZ data

optimise(IHS.loglik, lower=0.001, upper=100, x=UWdat$concentrationZ, maximum=TRUE)

## Set our best value for theta based on output max.theta above

best.theta <- 4.356475

## apply transformation to data

UWdat$trans.concentrationZ <- asinh(best.theta * UWdat$concentrationZ)/best.theta

# Fit Full model (here, point = section)

m1.Full <- lme(trans.concentrationZ~ element, random = ~1|ID/strand/layer/point, UWdat)

plot(m1.Full)

qqnorm(m1.Full,abline=c(0,1))

hist(resid(m1.Full))

summary(m1.Full)

anova(m1.Full)

r.squaredGLMM(m1.Full)

# Full model minus random factor 'point'

m1.noPoint <- lme(trans.concentrationZ~ element, random = ~1|ID/strand/layer, UWdat)

summary(m1.noPoint)

anova(m1.noPoint)

r.squaredGLMM(m1.noPoint)

# compare models

anova(m1.Full,m1.noPoint) *# No difference, use ‘* m1.noPoint’

# m1.noPoint minus layer

m1.noLayer <- lme(trans.concentrationZ~ element, random = ~1|ID/strand, UWdat)

summary(m1.noLayer)

anova(m1.noLayer)

r.squaredGLMM(m1.noLayer)

# compare models

anova(m1.noPoint,m1.noLayer) *# Layer must be kept in*

# m1.noPoint minus random factor 'strand'

m1.noStrand <- lme(trans.concentrationZ~ element, random = ~1|ID/layer, UWdat)

summary(m1.noStrand)

anova(m1.noStrand)

r.squaredGLMM(m1.noStrand)

# compare models

anova(m1.noPoint,m1.noStrand) *# Strand must be kept in*

# m1.noPoint minus random factor 'ID'

m1.noID <- lme(trans.concentrationZ~ element, random = ~1|strand/layer, UWdat)

summary(m1.noID)

anova(m1.noID)

r.squaredGLMM(m1.noID)

# compare models

anova(m1.noPoint,m1.noID) *# ID must be kept in*

#### Text S1.2 R-code, analyses of cleaning-layer effects subset by element

## Load libraries

library(nlme)

library(geoR)

library(multcomp)

library(MuMIn)

## Re-level so that ‘intercept’ in the LMEs is the ‘interior’ and/or ‘unwashed’

data$CleanLayer <- relevel(data$CleanLayer, ref = "UWinterior")

## Fit a model 3 with ‘method’ and Participant ID as random factor

## Plot & check distribution, transform ‘Element’ if required (see Text S1.1)

## Calculate post-hoc Tukey comparisons

m3 <- lme(element~ CleanLayer, random = ~1|ID/strand, data)

plot(m3)

qqnorm(m3,abline=c(0,1))

hist(resid(m3))

summary(m3)

anova(m3)

r.squaredGLMM(m3)

comp.cleanlayer<-glht(m3, linfct=mcp(CleanLayer="Tukey"))

print(summary(comp.cleanlayer))

#### Text S1.3 R-code, analyses of cleaning effects

# Load packages

library(nlme)

library(geoR)

library(multcomp)

library(MuMIn)

#  Re-level so that “intercept”  interior values & subset to unwashed data only

dat$layer <- relevel(dat$layer, ref = "interior")

dat$cleaning <- relevel(dat$cleaning, ref = "UW")

## Plot & check distribution, transform ‘Element’ if required (see Text S1.1)

## Optimise for concentrationZ data

optimise(IHS.loglik, lower=0.001, upper=100, x=dat$concentrationZ, maximum=TRUE)

## Set our best value for theta based on output max.theta above

best.theta <- 2.702507

## apply transformation data

dat$trans.concentrationZ <- asinh(best.theta * dat$concentrationZ)/best.theta

# Fit Full model

m2.Full <- lme(trans.concentrationZ~ element*layer*cleaning, random = ~1|ID/strand/point, dat)

plot(m1.Full)

qqnorm(m1.Full,abline=c(0,1))

hist(resid(m1.Full))

r.squaredGLMM(m2.Full)

# Full model minus random factor 'point' (ie. section)

m2.noPoint <- lme(trans.concentrationZ~ element*layer*cleaning, random = ~1|ID/strand, dat)

r.squaredGLMM(m2.noPoint)

anova(m2.Full,m2.noPoint) # No difference, use ‘m2.noPoint’

# m1.noPoint minus random factor 'strand'

m2.noStrand <- lme(trans.concentrationZ~ element*layer*cleaning, random = ~1|ID, dat)

r.squaredGLMM(m2.noStrand)

anova(m2.noPoint,m2.noStrand) *# Strand must be kept in*

# m1.noPoint minus random factor 'ID'

m2.noID <- lme(trans.concentrationZ~ element*layer*cleaning, random = ~1|strand, dat)

r.squaredGLMM(m2.noID)

anova(m2.noPoint,m2.noID) *# ID must be kept in*

# get summary data from best model (m2.noPoint)

m2.noPoint <- lme(trans.concentrationZ~ element*layer*cleaning, random = ~1|ID/strand, dat)

anova(m2.noPoint)

r.squaredGLMM(m2.noPoint)

## **Supplementary Table S1.** Analyte list and operating parameters

| ICP-MS Plasma Conditions | |
| --- | --- |
| RF Power | 1350 W |
| RF Matching | 1.3 V |
| Sample Depth | 4.5 mm |
| Nebulizer Gas | 0.90 L/min |
| Total sampling period | 0.4778 s |

| ICP-MS Elements Dwell Time | | | |
| --- | --- | --- | --- |
| Element | Duration | Element | Duration |
| ^27^Al | 0.02 s | ^63^Cu | 0.02 s |
| ^31^P | 0.02 s | ^66^Zn | 0.02 s |
| ^43^Ca | 0.02 s | ^75^As | 0.02 s |
| ^53^Cr | 0.02 s | ^88^Sr | 0.02 s |
| ^55^Mn | 0.03 s | ^111^Cd | 0.02 s |
| ^57^Fe | 0.02 s | ^137^Ba | 0.02 s |
| ^59^Co | 0.02 s | ^201^Hg | 0.02 s |
| ^60^Ni | 0.02 s | ^208^Pb | 0.02 s |

## **Supplementary Table S2.** Sources of variation in unwashed hair

| Model | | M1 | M2 | M3 | M4 | M5 |
| --- | --- | --- | --- | --- | --- | --- |
|  | AIC | 4864 | 4862 | 5310 | 5194 | 8677 |
|  | *c*R^2^ | 0.282 | 0.282 | 0.257 | 0.259 | 0.077 |
|  | *m*R^2^ | 0.068 | 0.068 | 0.068 | 0.068 | 0.068 |
|  |  |  |  |  |  |  |
| Random nested factors (Standard Deviation) | | | | | | |
|  | individual | 0.13 | 0.13 | 0.13 | 0.12 | − |
|  | strand | 0.04 | 0.04 | 0.06 | − | 0.00 |
|  | layer | 0.04 | 0.07 | − | 0.07 | 0.03 |
|  | section | 0.00 | − | − | − | − |
|  | residual | 0.28 | 0.28 | 0.28 | 0.28 | 0.31 |
|  |  |  |  |  |  |  |
| Fixed factor (element) | | |  |  |  |  |
|  | numerator DF | 15 | 15 | 15 | 15 | 15 |
|  | denominator DF | 15495 | 16421 | 16475 | 16491 | 16523 |
|  | F-value | 105 | 105 | 101 | 102 | 82 |
|  | *P*-value | <0.0001 | <0.0001 | <0.0001 | <0.0001 | <0.0001 |
|  |  |  |  |  |  |  |
| Model comparison | |  |  |  |  |  |
|  | models |  | M1 v M2 | M2 v M3 | M2 v M4 | M2 v M5 |
|  | DF |  | 21, 20 | 20, 19 | 20, 19 | 20, 19 |
|  | LR |  | <0.0001 | 450 | 334 | 3817 |
|  | *P* -value |  | 0.9971 | <0.0001 | <0.0001 | <0.0001 |
|  | Δ *c*R^2^ |  | 0.000 | 0.025 | 0.023 | 0.204 |
| **Linear mixed effects models** fit by REML; **Data**: IHS transformed (standardised concentrations for each element), Line scans of entire unwashed hair strands, n_observations_ = 16544, n_ID_ = 19, n_strand_ = 54, n_layer_ = 108, n_point_ = 1034. | | | | | | |
| **AIC** (Akaike Information Criterion), ***c*R^2^** (conditional r-squared, proportion of variance explained by both fixed & random factors), ***m*R^2^** (marginal r-squared, proportion of variance explained by fixed factor alone), **DF** = degrees of freedom, **Δ *c*R^2^** = *c*R^2^ (model 1) - *c*R^2^ (model 1), **LR** (likelihood ratio). | | | | | | |
| **M1**. Full (m1.Full): Concentration ~ element, random = ~1\|individual/strand/layer/section | | | | | | |
| **M2**. Full minus section (m1.noPoint): Concentration ~ element, random = ~1\|individual/strand/layer | | | | | | |
| **M3**. Full minus layer & section (m1.noLayer): Concentration ~ element, random = ~1\|individual/strand | | | | | | |
| **M4**. Full minus strand & section (m1.noStrand): Concentration ~ element, random = ~1\|individual/layer | | | | | | |
| **M5**. Full minus ID & section (m1.noID): Concentration ~ element, random = ~1\|strand/layer | | | | | | |

##

## **Supplementary Table S3.** Comparisons between pre-cleaning-layer element concentrations

| Element | mR^2^ | cR^2^ | UW^E^ v **UW^C^** | | EC^C^ v **UW^C^** | | EC^E^ v **UW^C^** | | NC^C^ v **UW^C^** | | NC^E^ v **UW^C^** | | UW^E^ v EC^C^ | | UW^E^ v EC^E^ | | UW^E^ v NC^C^ | | UW^E^ v NC^E^ | | EC^E^ v EC^C^ | | NC^C^ v EC^C^ | | NC^E^ v EC^C^ | | NC^C^ v EC^E^ | | NC^E^ v EC^E^ | | NC^E^ v NC^C^ | |
| --- | --- | --- | --- | --- | --- | --- | --- | --- | --- | --- | --- | --- | --- | --- | --- | --- | --- | --- | --- | --- | --- | --- | --- | --- | --- | --- | --- | --- | --- | --- | --- | --- |
| Al | 0.502 | 0.903 | 37.5 | * | 1.9 |  | 34.9 | * | -5.2 | * | 35.6 | * | 32.6 | * | -0.5 |  | 36.9 | * | -3.8 | * | 43.1 | * | -7.3 | * | 35.6 | * | -39.3 | * | 3.6 | * | 54.9 | * |
| As | 0.118 | 0.363 | 5.9 | * | 0.1 |  | 9.2 | * | 2.5 |  | 9.9 | * | 5.8 | * | -3.3 | * | 3.1 | * | -4.3 | * | 10.3 | * | 2.5 |  | 10.3 | * | -6.6 | * | 1.2 |  | 8.3 | * |
| Ba | 0.171 | 0.670 | 1.2 |  | -4.4 | * | -11.6 | * | -15.9 | * | -7.9 | * | 5.5 | * | 12.7 | * | 17.0 | * | 8.9 | * | -8.9 | * | -12.4 | * | -3.9 | * | -5.2 | * | 3.3 | * | 10.0 | * |
| Ca | 0.293 | 0.541 | 4.6 | * | -4.5 | * | 0.6 |  | -18.6 | * | 2.2 |  | 9.1 | * | 4.0 | * | 23.0 | * | 2.3 |  | 5.7 | * | -14.8 | * | 6.8 | * | -20.0 | * | 1.6 |  | 22.8 | * |
| Cd | 0.145 | 0.522 | -5.1 | * | -2.7 |  | -12.4 | * | -10.6 | * | -10.1 | * | -2.1 |  | 7.6 | * | 6.1 | * | 5.6 | * | -12.1 | * | -8.5 | * | -8.0 | * | 1.1 |  | 1.5 |  | 0.6 |  |
| Co | 0.123 | 0.738 | 4.6 | * | 4.2 | * | 10.8 | * | -2.1 |  | 11.4 | * | -0.1 |  | -6.7 | * | 5.8 | * | -7.7 | * | 9.1 | * | -6.2 | * | 8.0 | * | -12.4 | * | 1.7 |  | 19.6 | * |
| Cr | 0.247 | 0.585 | -7.4 | * | 6.7 | * | 8.3 | * | 9.0 | * | 12.7 | * | -13.7 | * | -15.2 | * | -15.6 | * | -19.2 | * | 2.0 |  | 2.8 | * | 6.7 | * | 1.3 |  | 5.1 | * | 4.6 | * |
| Cu | 0.447 | 0.857 | 9.8 | * | 4.3 | * | 9.6 | * | -14.9 | * | -24.8 | * | 4.4 | * | -0.9 |  | 22.7 | * | 32.5 | * | 7.3 | * | -19.7 | * | -30.0 | * | -24.6 | * | -35.0 | * | -14.4 | * |
| Fe | 0.409 | 0.834 | 22.6 | * | 4.4 | * | 29.3 | * | -1.7 |  | 30.6 | * | 17.3 | * | -7.7 | * | 22.2 | * | -10.1 | * | 30.4 | * | -6.1 | * | 27.7 | * | -30.9 | * | 2.9 | * | 39.5 | * |
| Hg | 0.006 | 0.903 | 6.1 | * | 4.8 | * | 3.0 | * | 0.5 |  | 1.2 |  | 1.1 |  | 2.9 | * | 5.1 | * | 4.4 | * | -2.2 |  | -4.2 | * | -3.5 | * | -2.4 |  | -1.7 |  | 0.9 |  |
| Mn | 0.192 | 0.741 | 3.4 | * | -5.3 | * | -6.6 | * | -20.2 | * | -6.5 | * | 8.5 | * | 9.8 | * | 23.3 | * | 9.5 | * | -1.7 |  | -16.0 | * | -1.6 |  | -14.6 | * | -0.2 |  | 16.9 | * |
| Ni | 0.305 | 0.746 | 20.6 | * | 0.3 |  | 9.5 | * | -8.3 | * | 7.2 | * | 18.0 | * | 8.8 | * | 24.9 | * | 9.3 | * | 12.5 | * | -9.1 | * | 7.3 | * | -17.8 | * | -1.5 |  | 22.3 | * |
| P | 0.164 | 0.694 | -13.2 | * | 1.9 |  | -2.4 |  | 1.2 |  | 7.4 | * | -14.0 | * | -9.6 | * | -12.2 | * | -18.5 | * | -5.6 | * | -0.6 |  | 6.0 | * | 3.5 | * | 10.1 | * | 8.6 | * |
| Pb | 0.271 | 0.729 | -3.9 | * | 0.1 |  | -7.0 | * | -20.6 | * | -11.9 | * | -3.6 | * | 3.5 | * | 17.4 | * | 8.7 | * | -9.5 | * | -21.7 | * | -12.6 | * | -14.9 | * | -5.8 | * | 12.2 | * |
| Sr | 0.208 | 0.730 | -0.9 |  | -4.8 | * | -16.8 | * | -16.4 | * | -12.7 | * | 4.0 | * | 15.9 | * | 15.6 | * | 12.0 | * | -15.5 | * | -12.6 | * | -8.7 | * | -0.9 |  | 3.0 | * | 4.9 | * |
| Zn | 0.484 | 0.811 | -8.2 | * | -2.3 |  | -21.9 | * | -27.0 | * | -29.3 | * | -5.2 | * | 14.4 | * | 20.2 | * | 22.5 | * | -26.3 | * | -26.2 | * | -28.6 | * | -7.4 | * | -9.7 | * | -3.2 | * |
| **Post-hoc Tukey contrasts** (with single-step adjusted p-values for multiple comparisons), Linear mixed effects models fit by REML, Element~CleanLayer, Random factors = *strand* nested in *ID*. Data: Line scans of first 2cm of hair strands, n_observations_ = 1336, n_ID_ = 20, n_strand_ = 651; *m*R^2^ (marginal R-squared, proportion of variance explained by fixed factor alone), *c*R^2^ (conditional R-squared, proportion of variance explained by both fixed & random factors); Pre-cleaning treatment & layer, Un-Washed External (UW^E^), Un-Washed Interior (**UW^C^**), Ethanol External (EC^E^), Ethanol Interior (EC^C^),Nitric External (NC^E^), Nitric Interior (NC^C^); *z*-values displayed with **P* <0.05. | | | | | | | | | | | | | | | | | | | | | | | | | | | | | | | | |

## **Supplementary Table S4.** Element, pre-cleaning and layer effects on hair

| Best fit model | numDF | F-value | *P*-value |
| --- | --- | --- | --- |
| (intercept) | 1 | 15 | 0.0003 |
| element | 15 | 13 | <0.0001 |
| layer | 1 | 402 | <0.0001 |
| cleaning | 2 | 35 | <0.0001 |
| element:layer | 15 | 137 | <0.0001 |
| element:cleaning | 30 | 86 | <0.0001 |
| layer:cleaning | 2 | 137 | <0.0001 |
| element:layer:cleaning | 30 | 17 | <0.0001 |
| **Linear mixed effects model fit by REML**; Random factors = strand nested in individual. Data: IHS transformed (standardised concentrations for each element), Line scans of first 2cm of hair strands, denominator DF = 20630, n_observations_ = 21376, n_ID_ = 20, n_strand_ = 651; AIC = 14492, *c*R^2^ =0.351, *m*R^2^ = 0.195, numDF (numerator DF). | | | |

## **Supplementary Table S5.** Correlations among pre-cleaning and analyses of hair samples

|  | Pre-cleaning & Layer | | | | | | | |
| --- | --- | --- | --- | --- | --- | --- | --- | --- |
|  |  |  | UW | UW | EC | EC | NC | NC |
| Element | 𝜆 | % | interior | ext' | interior | ext' | interior | ext' |
| Al | 4.3 | 72 | 0.38 | 0.43 | 0.38 | 0.37 | 0.45 | 0.44 |
| As | 2.0 | 34 | 0.46 | 0.10 | 0.59 | 0.39 | -0.01 | 0.53 |
| Ba | 3.4 | 57 | 0.51 | 0.46 | 0.43 | 0.31 | 0.46 | 0.18 |
| Ca | 3.1 | 52 | 0.52 | 0.51 | 0.40 | -0.09 | 0.52 | 0.18 |
| Cd | 3.5 | 58 | 0.52 | -0.05 | 0.48 | -0.09 | -0.48 | -0.51 |
| Co | 2.8 | 47 | 0.51 | 0.38 | 0.27 | -0.03 | 0.51 | 0.51 |
| Cr | 3.0 | 50 | 0.44 | 0.44 | -0.43 | -0.41 | 0.37 | -0.36 |
| Cu | 4.1 | 68 | 0.46 | 0.45 | 0.42 | 0.34 | 0.39 | 0.37 |
| Fe | 4.3 | 71 | 0.36 | 0.39 | 0.43 | 0.38 | 0.46 | 0.42 |
| Hg | 5.3 | 89 | 0.43 | 0.43 | 0.42 | 0.38 | 0.41 | 0.37 |
| Mn | 3.7 | 62 | 0.48 | 0.46 | 0.42 | 0.31 | 0.44 | 0.29 |
| Ni | 3.1 | 52 | 0.54 | 0.29 | 0.47 | 0.27 | 0.41 | 0.40 |
| P | 3.5 | 58 | 0.39 | 0.02 | 0.49 | 0.39 | 0.48 | 0.47 |
| Pb | 2.8 | 46 | 0.48 | 0.50 | 0.49 | 0.49 | 0.07 | -0.19 |
| Sr | 3.4 | 57 | 0.52 | 0.50 | 0.30 | 0.15 | 0.43 | 0.42 |
| Zn | 2.5 | 41 | 0.46 | 0.52 | 0.42 | 0.32 | 0.37 | 0.32 |

**Principal Components Analysis** (PCA) on correlations, among methods for each element (data = individual means taken across strand-medians from 2cm line scans, subset by element); 𝜆 (1st eigenvalue), % (percent of variance explained by first eigenvector, PC1), PC1 loadings (colour & shade indicates similar loading); interior (interior layer), ext' (external layer), UW (un-washed), EC (ethanol pre-cleaned), NC (nitric acid pre-cleaned).


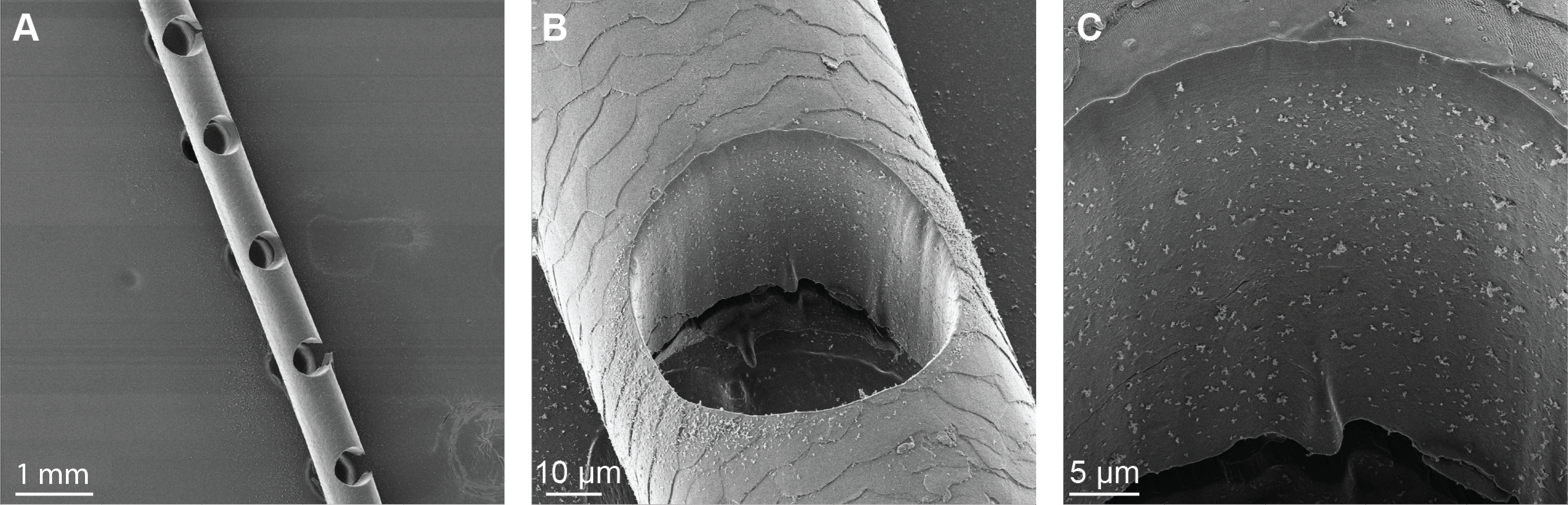


## **Supplementary** Figure S6. HIM images of LA-ICP-MS spot drilling. Zeiss helium ion microscope images of a hair strand with spot drilling at increasing magnification: A) 1mm, B) 10μm, and C) 5μm. Photo credit: Dr Peter Hines (Central Analytical Research Facility operated by the Institute for Future Environments at Queensland University of Technology).


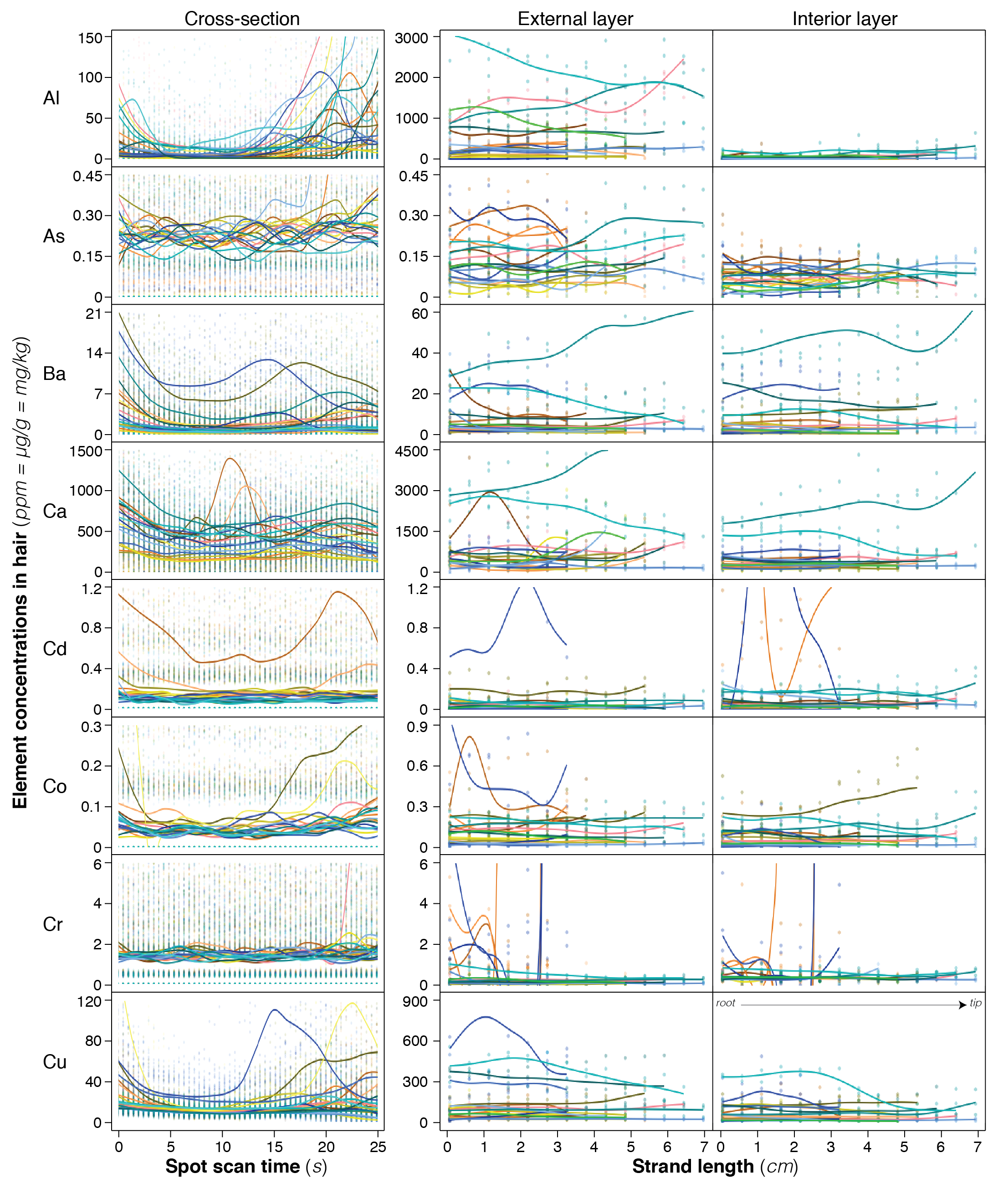


#### Supplementary Figure S7. Variation in Al, As, Ba, Ca, Cd, Co, Cr and Cu of unwashed hair concentrations from LA-ICP-MS. Element concentration plotted across (spot drilling, cross-section, time in seconds as a proxy for depth, n = 20) and along (line scans, external & interior layers, n = 19) hair strands. Raw spot data (~0.3s instantaneous concentrations) and averaged line scan data (mean across 0.5cm sections) from every hair strand are plotted, and overlaid by a smoothed cubic spline across strands for each individual (curvy lines, 𝜆 = 0.05). Each individual is delineated by a unique colour.

####
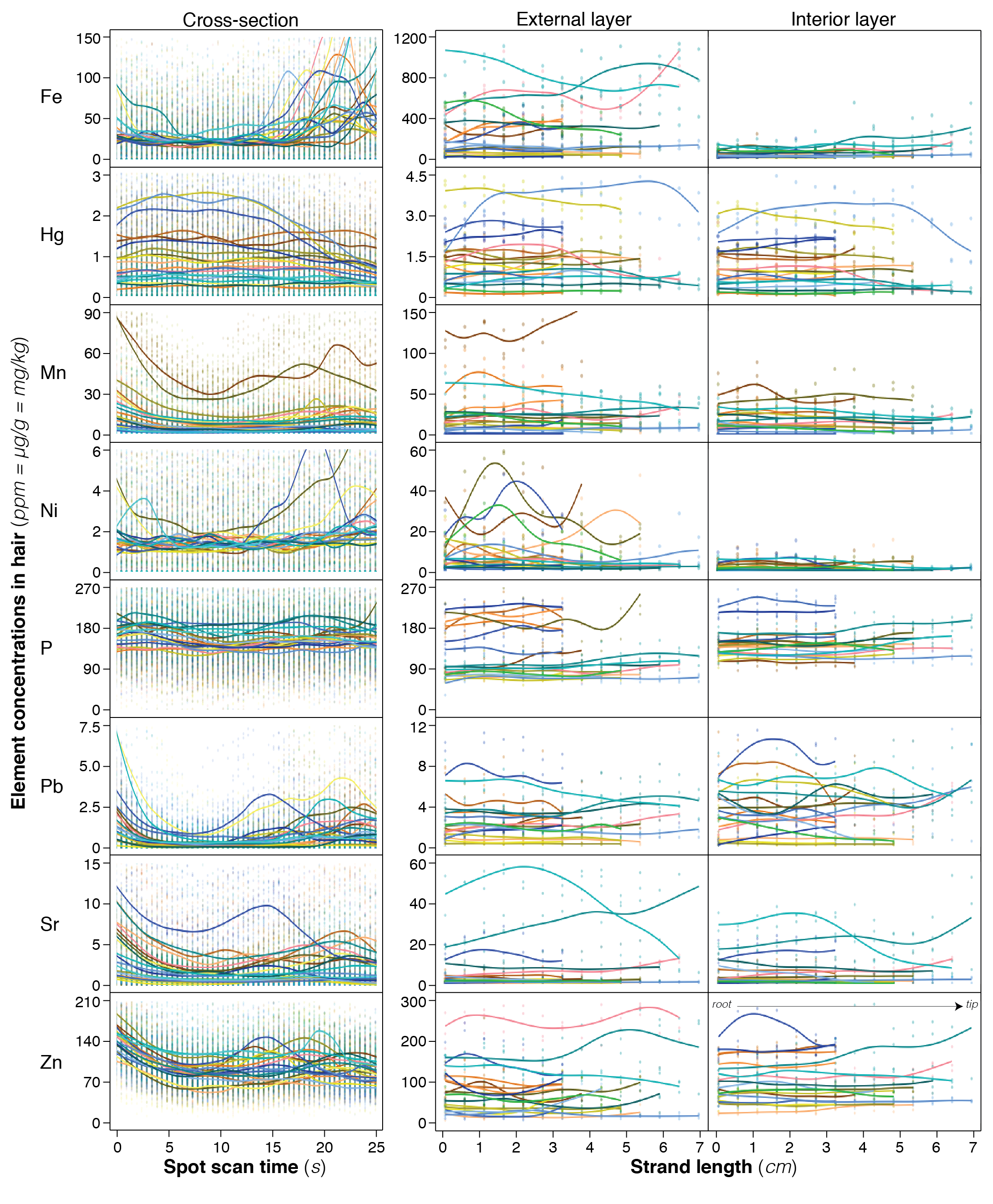


## **Supplementary Figure S8. Variation in Fe, Hg, Mn, Ni, P, Pb, Sr and Zn of unwashed hair concentrations from LA-ICP-MS.** Element concentration plotted across (spot drilling, cross-section, time in seconds as a proxy for depth, n = 20) and along (line scans, external & interior layers, n = 19) hair strands. Raw spot data (~0.3s instantaneous concentrations) and averaged line scan data (mean across 0.5cm sections) from every hair strand are plotted, and overlaid by a smoothed cubic spline across strands for each individual (curvy lines, 𝜆 = 0.05). Each individual is delineated by a unique colour.


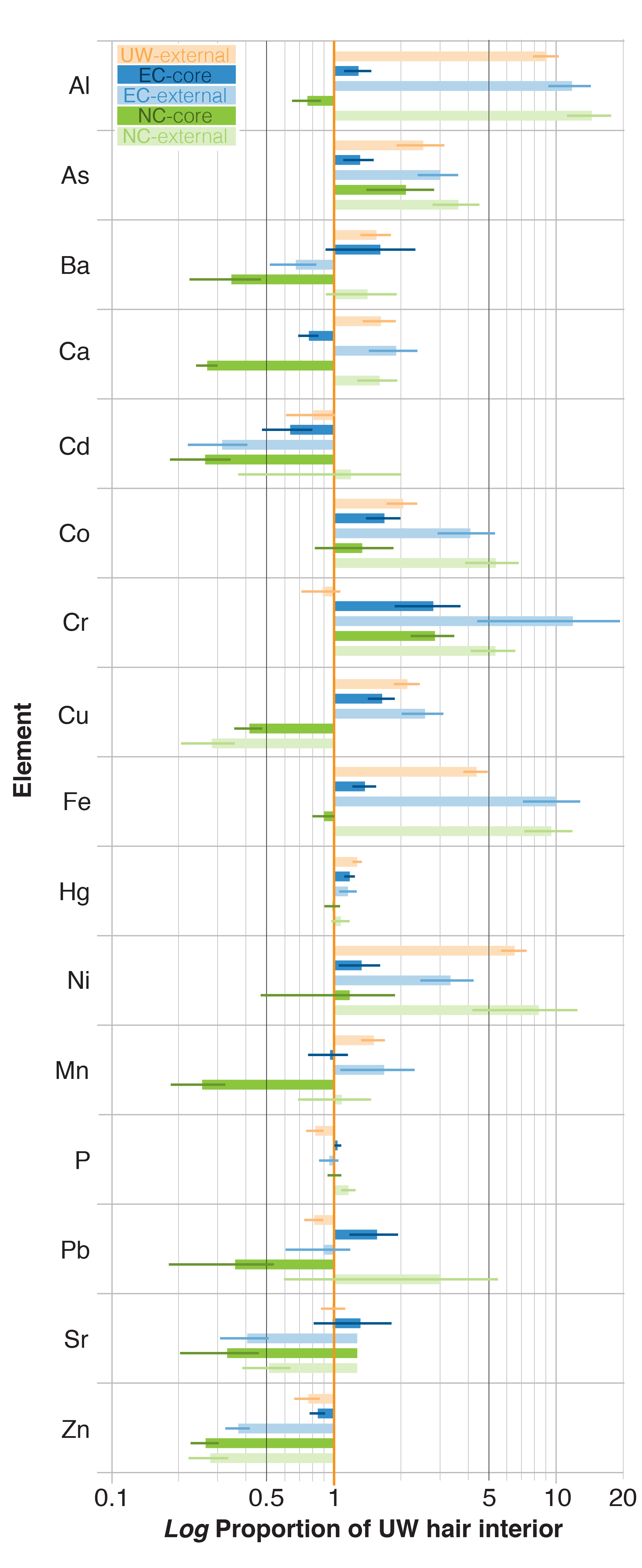


## **Supplementary Figure S9. Pre-cleaned hair relative to unwashed interior concentrations**. LA-ICP-MS element concentrations of five LA-ICP-MS measures as a proportion of unwashed interior values (UW interior, dark orange vertical line). Five other LA-ICP-MS measures: unwashed external layer (light orange bars), ethanol-pre-cleaned interior and external layers (EC, dark & light blue bars), nitric acid-washed interior and external layers (NC, dark & light green bars). Data displayed are mean and standard error across individuals (2cm line scans: individual mean across strand-medians). Note logarithmic scale.


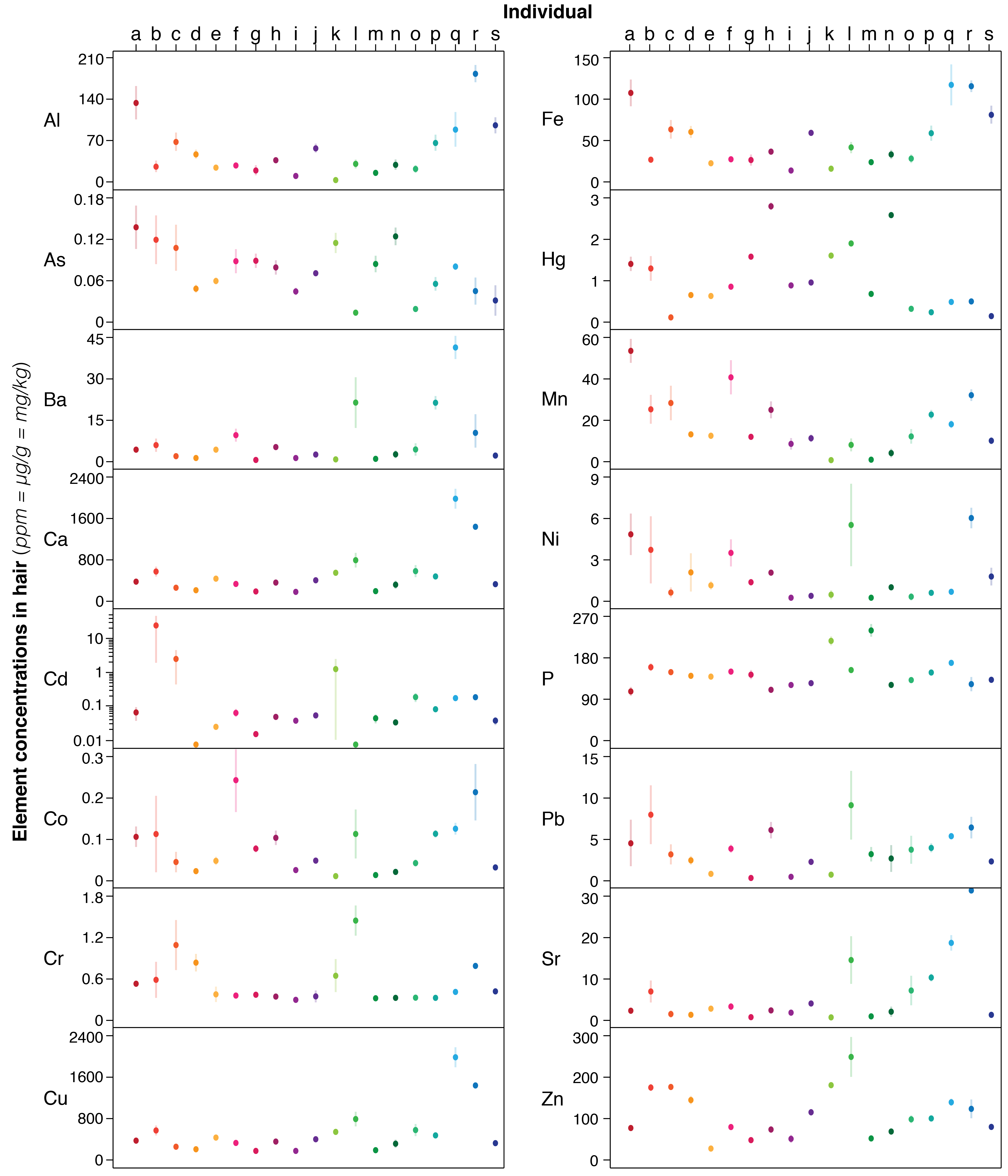


## **Supplementary Figure S10.Individual variation in interior element concentrations of unwashed hair**. Mean concentrations (solid circles) and standard errors (vertical lines) of elements for 19 individuals from LA-ICP-MS line scans of the 1st 2cm interior layer of unwashed hair strands. Data are individual means calculated across 3 strand-means (2 strands for individuals k, l & m). Note for Cd the logarithmic scale and zero values for individuals f & n.
